# Athlete's Heart: A Cardiovascular Step-By-Step Multimodality Approach

**DOI:** 10.31083/j.rcm2405151

**Published:** 2023-05-19

**Authors:** Stefano Palermi, Elena Cavarretta, Flavio D’Ascenzi, Silvia Castelletti, Fabrizio Ricci, Marco Vecchiato, Alessandro Serio, Luna Cavigli, Eduardo Bossone, Giuseppe Limongelli, Alessandro Biffi, Emanuele Monda, Andre La Gerche, Aaron Baggish, Antonello D’Andrea

**Affiliations:** ^1^Public Health Department, University of Naples Federico II, 80131 Naples, Italy; ^2^Department of Medical-Surgical Sciences and Biotechnologies, Sapienza University of Rome, 04100 Latina, Italy; ^3^Mediterranea Cardiocentro, 80122 Naples, Italy; ^4^Department of Medical Biotechnologies, Division of Cardiology, University of Siena, 53100 Siena, Italy; ^5^Cardiology Department, Istituto Auxologico Italiano IRCCS, 20149 Milan, Italy; ^6^Department of Neuroscience, Imaging and Clinical Sciences, G.d’Annunzio University of Chieti-Pescara, 66100 Chieti, Italy; ^7^Sports and Exercise Medicine Division, Department of Medicine, University of Padova, 35128 Padova, Italy; ^8^Division of Cardiology, AORN A Cardarelli Hospital, 80131 Naples, Italy; ^9^Department of Translational Medical Sciences, University of Campania Luigi Vanvitelli, 80131 Naples, Italy; ^10^Med-Ex, Medicine & Exercise, Medical Partner Scuderia Ferrari, 00187 Rome, Italy; ^11^Department of Translational Medical Sciences, University of Campania Luigi Vanvitelli, 80131 Naples, Italy; ^12^Clinical Research Domain, Baker Heart and Diabetes Institute, Melbourne, VIC 3004, Australia; ^13^Cardiovascular Performance Program, Massachusetts General Hospital, Boston, MA 02114, USA; ^14^Unit of Cardiology and Intensive Coronary Care, Umberto I Hospital, 84014 Salerno, Italy

**Keywords:** athlete's heart, cardiovascular imaging, pre-participation screening, sports activity, sports cardiology, sudden cardiac death

## Abstract

“Athlete’s heart” is a spectrum of morphological, functional, and regulatory changes that occur in people who practice regular and long-term intense physical activity. The morphological characteristics of the athlete’s heart may overlap with some structural and electrical cardiac diseases that may predispose to sudden cardiac death, including inherited and acquired cardiomyopathies, aortopathies and channelopathies. Overdiagnosis should be avoided, while an early identification of underlying cardiac life-threatening disorders is essential to reduce the potential for sudden cardiac death. A step-by-step multimodality approach, including a first-line evaluation with personal and family history, clinical evaluation, 12-lead resting electrocardiography (ECG), followed by second and third-line investigations, as appropriate, including exercise testing, resting and exercise echocardiography, 24-hour ECG Holter monitoring, cardiac magnetic resonance, computed tomography, nuclear scintigraphy, or genetic testing, can be determinant to differentiate between extreme physiology adaptations and cardiac pathology. In this context, cardiovascular imaging plays a key role in detecting structural abnormalities in athletes who fall into the grey zone between physiological adaptations and a covert or early phenotype of cardiovascular disease.

## 1. Introduction 

Physical activity, defined as any body movement resulting from the contraction of skeletal muscle that raises energy expenditure above the resting metabolic rate [1], if carried out regularly and for long periods, can result in substantial adaptations of the cardiovascular (CV) system to improve athletic performance. The athlete’s heart results from these morphological, functional and regulatory adaptations and may be characterized by increased mass, cavity dimensions, and wall thickness with at least normal systolic and diastolic function [2, 3, 4, 5, 6]. The physiological factors of this remodeling are various and not fully known, but they depend on many non-modifiable properties of the athletes and the type of exercise, including type and duration of physical activity, other than environmental and genetic factors.

Sometimes, there may be some overlap (the so-called “grey zone”) between the physiological adaptation of the athlete’s heart and some pathological conditions, such as hypertrophic cardiomyopathy (HCM) or arrhythmogenic cardiomyopathy (ACM), that may pose an athlete at risk of dying suddenly. Therefore, the differentiation between physiological and pathological cardiac anomalies in athletes may be challenging, but it is mandatory because the incorrect diagnosis may have important consequences, such as exclusion from competitive sport, false reassurance, and missed opportunities for effective therapeutic interventions. Sudden cardiac death (SCD) in young athletes is usually caused by genetic or congenital structural cardiac disorders [7, 8], such as HCM, ACM, or an anomalous coronary artery origin. In athletes >35 years of age, most of all SCDs are due to atherosclerotic coronary artery disease (CAD) [9].

For this reason, pre-participation cardiovascular screening (PPS) aims to identify pathological conditions in athletes to prevent morbidity and SCD [10, 11, 12]. However, the best strategies remain controversial [12]: while European [13] guidelines recommend performing a 12-lead resting electrocardiogram (ECG) and Italian [14] guidelines even also a mandatory exercise stress test (EST) as the initial screening of competitive athletes, the United States [10] and American Heart Association [2] positions do not support a systematic national screening based on resting ECG in competitive athletes. However, both agree that further evaluations should be recommended in symptomatic (syncope, chest pain, exercise dyspnea, palpitations) and/or high CV-risk patients [8, 15, 16]. Therefore, the PPS of asymptomatic competitive or leisure athletes must be distinguished from the assessment of athletes reporting specific symptoms or conditions or conditions that may fall into grey zones [3].

To date, many cardiovascular diagnostic techniques have been tested on athletes, but the best strategies to highlight the main features of the athlete’s heart remain unknown [17]. Therefore, the present paper summarizes evidence about a step-by-step CV multimodality approach to diagnosing the athletes’ heart.

## 2. Physiological and Pathological Cardiac Adaptations to Physical Activity

Systematic training leads to CV changes that markedly increase cardiorespiratory fitness, enabling the athlete to improve performance and achieve higher sports results. The CV system can significantly adapt to changes in the hemodynamic conditions of the body [18]. The perfect efficiency of the CV system is therefore crucial for physical performance: the greater supply of oxygen to the muscles is ensured by increased district blood flow and an increased oxygen extraction from blood [19]. Maximal oxygen uptake (${\mathrm{VO}}_{2}$max) is a physiological characteristic determined by the product of maximal cardiac output (the product of heart rate and left ventricle stroke volume) and maximal arteriovenous oxygen content difference [20].

Also, sports activity is associated with variations in the overall hemodynamic state. Endurance and strength training lead the athlete’s heart to different types of adaptations, even though most disciplines cause mixed adaptation scenarios. The persistence of such modifications in athletes depends on various factors, such as sex, age, ethnicity and physiological characteristics of the subject [21, 22], which are largely genetically determined: indeed female [21] and pediatric [23, 24] athlete’s hearts are growing topic in current literature. Furthermore, these adaptations vary on the duration, type and intensity of the sports activity practiced by the subject [20, 25]. Studies suggest that at least 3 hours of training per week for at least 3 months could be sufficient to see some initial morpho-functional adaptations of the heart [20], but identifying an athlete’s heart requires much more training. Endurance activity can be defined as aerobic isotonic dynamic exercise: it involves large muscle groups working thanks to aerobic metabolism and includes sporting disciplines such as long and middle-distance running, swimming or cycling [8]. Strength activity can be defined as >30% maximal voluntary contraction and includes sporting disciplines performed at high intensity unsustainable by oxygen delivery alone and requiring metabolism of stored energy to be processed largely by glycolysis: examples are martial arts, short running distance, wind-surfing and weight-lifting. It is important to note that many sporting disciplines involve a combination of strength and endurance exercises (football, basketball, volleyball) and, therefore, there is likely to be an overlap in ranges [26]. In 1975, Morganroth *et al*. [27] introduced the concept that endurance and strength forms of exercise lead to different adaptations in cardiac structure [28]. Specifically, athletes exposed to endurance training demonstrate eccentric left ventricle (LV) hypertrophy, often accompanied by a right ventricle (RV) dilatation, due to an increased LV volume that increases diastolic wall stress [20]. Athletes exposed to strength training instead demonstrate concentric LV hypertrophy, characterized by normal LV cavity dimensions, but increased wall thickness and mass because of a pressure overload and increased systolic wall stress [20]. This hypothesis, called the “Morganroth Hypothesis” from the name of the scientist who developed it, has some limitations because many sports, such as rowing or cycling, imply both endurance and strength exercise, and hypertrophy results in an intermediate phenotype [8]. Moreover, this hypothesis has been challenged by recent studies suggesting that the increase in LV mass is proportional to the increase in LV volume (balanced remodeling) irrespective of the sports discipline [29], and normal LV geometry can be frequently observed also in top-level athletes [30].

Autonomic nervous system adjustments to the heart and blood vessels are necessary for mediating the CV responses required to meet the metabolic demands of working skeletal muscle during exercise; these demands are met by precise exercise intensity-dependent alterations in sympathetic and parasympathetic nerve activity [31]. Endurance training increases parasympathetic activity and decreases sympathetic activity in the heart at rest. These two training-induced autonomic effects, coupled with a possible reduction in intrinsic heart rate, decrease resting heart rate. Long-term endurance training also decreases submaximal exercise heart rate by reducing sympathetic activity to the heart [32]. However, the athlete’s heart is also a proarrhythmic heart, which may explain the prevalence of atrial fibrillation, ventricular arrhythmias and conduction tissue disease in athletes: dilatation of atria and ventricles, hypertrophy, bradycardia, vagal tone at rest, ionic changes, early repolarization, sympathetic tone during exercise and high wall stress are all possible underlying mechanisms [33].

Furthermore, heat [34] and cold [35] adaptation, as well as high [36] and low [37] atmospheric pressure exposure during exercise [38], are equally responsible for different CV behaviour to increase athletic performance. Also, some drugs, approved for therapeutic use in some pathologies but used by athletes not only for their capacity to improve selective aspects of physical performance [39], but also as doping substances, lead to heart alterations which can have serious side effects, especially when used at high doses and for long duration: it is the case of anabolic androgenic steroids [40, 41, 42].

## 3. The Step-By-Step Approach to Athlete’s Heart

**Fig. 1.**
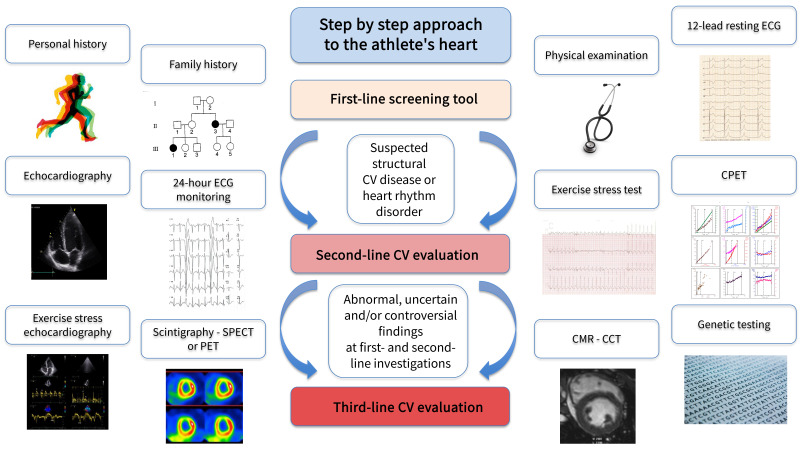
**The step-by-step approach in the management of athlete’s heart**. CV, cardiovascular; ECG, electrocardiogram; CPET, cardiopulmonary exercise test; CMR, cardiac magnetic resonance; CCT, cardiac computer tomography; SPECT, single photon emission computer tomography; PET, positron emission tomography.

To discriminate between extreme physiology adaptations or an early-stage structural cardiac disease is a crucial task for the physician evaluating an athlete. It is important to point out that the physician performing and/or interpreting an athlete’s PPS should possess a basic knowledge of fundamental exercise physiology and exercise-induced cardiac remodeling features, to avoid the misinterpretation of data [43]. We therefore propose a systematic approach to conducting the CV evaluation of an athlete. The optimal way to begin the PPS should include family and personal history collection, physical examination and 12-lead resting ECG, as proposed by several scientific societies and as shown as a first-line evaluation in our step-by-step approach (Fig. 1). Only if in the presence of clinical suspicion or ECG abnormalities, it may be necessary to request other examinations, as indicated in the International Recommendations for Electrocardiographic Interpretation in athletes [44]. In that sense, the most common, accessible and cost-effective exams as a second-line examination are echocardiography, EST, 24-hours Holter ECG monitoring and cardiopulmonary exercise testing (CPET). If the results of one or more of these second-line evaluations are highly suspicious or fall in the grey zone, a third-line evaluation is needed, which is represented by less accessible or more costly diagnostic techniques such as exercise stress echocardiography (ESE), cardiovascular magnetic resonance (CMR), coronary computer tomography (CCT), genetic testing, single photon emission computed tomography (SPECT) and positron emission tomography (PET).

### 3.1 First-Line Evaluation 

Even if today there are numerous advanced modalities to assess CV health, the backbones of the athlete’s screening process are family and personal history, including sports history and potential assessment of CV effect of doping substances or ergogenic aids, and physical examination. The American Heart Association recommends these as the only tools in PPS [2]. Several questionnaires exist about the family and personal history of the athlete, and they are all based on the detection of congenital or personal CV diseases that may pose the athletes at risk of SCD [12], while the physical examination of the athletes aims at identifying heart and vessels’ congenital abnormalities (i.e., cardiac murmur, peripheral pulses), and features associated with genetic conditions such as the Marfan syndrome [45]. However, if used alone, their false positive rate is high [46, 47], especially if compared with to the PPS with 12-lead ECG, that remains still the most stand-alone and recommended screening method for athletes. Therefore, the simultaneous use of history, physical examination and ECG as first-line screening tools in athletes is highly recommended.

#### Electrocardiogram

ECG is a simple, quick, cheap and non-invasive diagnostic technique [48], that provides a graphic recording of the electrical cardiac activity. It is nowadays widely used for CV screening, given its important role in reducing SCD rate [49], but its cost effectiveness, the need for experienced physicians to correctly interpret it and a high false positive rate are criticisms often moved about it [12, 50, 51]. ECG changes in athletes are common and usually reflect adaptive structural and electrical remodeling of the heart in response to regular training [52, 53, 54, 55, 56]. Furthermore, ECG adaptations may vary according to demographic characteristics, such as age, sex and ethnicity, as well as the type of sport and level of training. Based on the International Recommendations for ECG interpretation in athletes, which should be applied only to those exercising vigorously for at least 4–8 hours per week [44], ECG findings in athletes are classified as normal, abnormal and borderline (Table 1, Ref. [44]): if one abnormal or two borderline findings together are detected, further evaluation must be performed. However, some of these adaptive changes overlap with patterns reflective of underlying pathology. Accurate interpretation of the ECG in asymptomatic athletes is of paramount importance to avoid unnecessary further investigations (given the possibility of false positive findings of this technique [50, 51]) or sport disqualification, and prevent serious consequences, including SCD, in case of high-risk cardiovascular conditions. A proposal of a modified algorithm for ECG interpretation in children athletes has been recently hypothesized [57].

**Table 1.** **ECG findings in athletes based on the international criteria [44]**.

Normal ECG findings	Borderline ECG findings	Abnormal ECG findings
Sinus bradycardia or sinus arrhythmia	Left axis deviation	ST-T repolarization abnormalities (T-wave inversion, ST-segment depression)
First-degree AV block, Mobitz type 1 second-degree AV block	Left atrial enlargement	Pathological Q waves
Ectopic atrial or junctional escape rhythm	Right axis deviation	QRS ≥140 ms duration
Incomplete RBBB	Right atrial enlargement	Epsilon wave
Early repolarization/ST-segment elevation	Complete RBBB	Complete LBBB
Increased QRS voltage criteria for left or right ventricular hypertrophy		QT Abnormalities (Long and Short)
ST elevation followed by T-wave inversion V1–V4 in black athletes		Ventricular pre-excitation
T wave inversion V1–V3 in <age 16 years		Brugada type 1 pattern
		Profound sinus bradycardia <30 bpm
		PR interval ≥400 ms
		Mobitz type 2 second-degree AV block, third-degree AV block
		≥2 PVCs at rest
		Atrial tachyarrhythmias
		Ventricular arrhythmias

ECG, electrocardiogram; AV, atrioventricular; LBBB, left bundle branch block; PVC, premature ventricular contraction; RBBB, right bundle branch block.

### 3.2 Second-Line Evaluation

Most scientific societies worldwide do not recommend the echocardiogram as a screening modality in athletes, even if its use in the initial PPS is growing [15, 16], given its potential role in identifying CV abnormalities that can be undetected by ECG [58, 59]. However, nowadays, echocardiography is a very useful second-line diagnostic modality [15], when a suspicion of a structural CV disease is raised. On the other side, when a heart rhythm disorder is suspected, exercise-related CV diagnostic modalities are recommended: first, an EST, often followed by a 24-hours ECG Holter monitoring, allows for the investigation of the athlete’s CV system during physical effort [60]. Therefore, echocardiography, EST and 24-hours ECG Holter monitoring are often used together as second-line investigation tools, given their wide availability and low cost. When it is necessary to follow up with an athlete, the three examinations or a combination of them are very effective in identifying subtle changes over time [61, 62]. Nevertheless, also CPET can have an important role in the diagnostic process of athlete’s heart [63, 64], but it requires experienced personnel, it is expensive and time-consuming, limiting its wide dissemination and use in athletes to only some selected cases.

#### 3.2.1 Echocardiogram 

Due to its ability to provide information on cardiac morphology, function and hemodynamics, its low cost and wide and easy availability, the use of echocardiography in athlete’s evaluation is increasing [58, 65, 66, 67], also given that the low acoustic chest impedance of the athletic population makes it possible to obtain high-quality images [16].

**Fig. 2.**
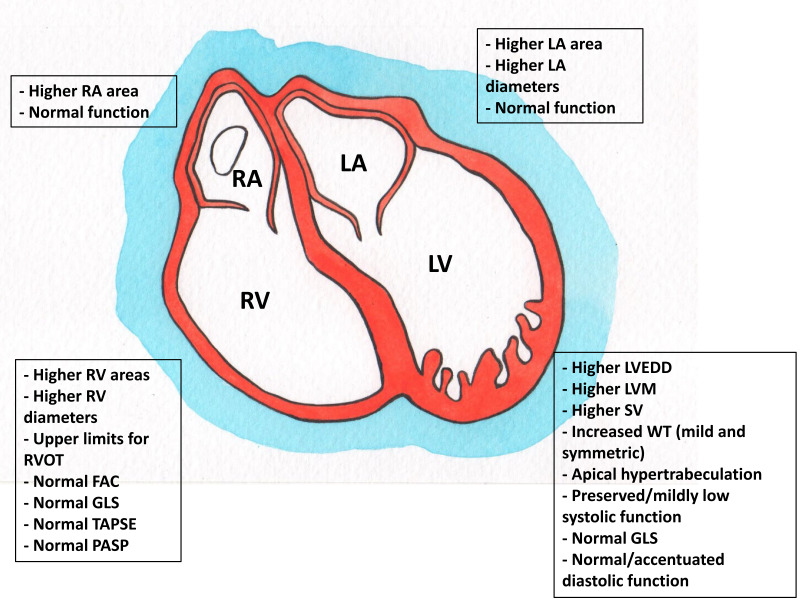
**Echocardiographic cardiovascular adaptations in the athlete’s heart**. RA, right atrium; LA, left atrium; RV, right ventricle; RVOT, right ventricle outflow tract; FAC, fractional area change; GLS, global longitudinal strain; TAPSE, tricuspid area plane systolic excursion; PASP, pulmonary artery systolic pressure; LV, left ventricle; LVEDD, left ventricular end-diastolic diameter; LVM, left ventricular mass; SV, stroke volume; WT, wall thickness.

Cardiovascular adaptations of an athlete’s heart include balanced increases in all heart chambers. While interest has largely focused on the LV in the past, attention has recently been directed to other structures such as the RV, the atria, and the aorta [68, 69]: adaptations to physical activity include a proportional increase in the left and right cardiac cavity sizes, increased LV wall thickness and LV mass, and supra-normal indices of systolic and diastolic function [70, 71, 72, 73] (Fig. 2). These adaptations, strictly dependent upon the duration, type and intensity of training, are often benign and physiological but may sometimes predispose to pathological conditions [74].

Several reference values about age, gender, ethnicity, and sports disciplines have been published in the literature by different study groups (Table 2, Ref. [75, 76, 77, 78, 79, 80]; Table 3, Ref. [80, 81]). However, we currently lack universally accepted cut-offs for basic echocardiographic measurements [75, 82], and therefore there are no unanimous recommendations about the use of echocardiographic cut-offs to distinguish between physiological and pathological adaptations. Indeed, comprehensive nomograms including sufficient sample size (of both genders), evaluating different ages (including master athletes) and ethnicities and various sports, evaluating a complete dataset of 2D (and new 3D and strain analysis indexes) echocardiographic measures, and built using a rigorous statistical approach (uniform normalization and way to express normalized data—preferably as Z-scores) are still missing in current literature [83]. Therefore, care is needed when interpreting this exam.

**Table 2.** **Athlete’s left heart echocardiography evaluation**.

Cardiac chamber	Parameter	Study	Mean value (± SD)
LV	EDD (mm) (BSA <1.8 M, BSA <1.5 F)	Boraita *et al*. [75], Spain – 2022 (3282 elite athletes, mean age 23 ± 6 years)	55 ± 4 M
			49 ± 4 F
		Magalski *et al*. [76], USA – 2011 (964 competitive athletes, ages 18–21 years)	52 ± 4 M
			46 ± 4 F
			49 ± 5 white
			50 ± 5 black
		Pelliccia *et al*. [77], Italy – 1991 (1309 elite athletes, mean age 22 years)	54.6 ± 3.5 high impact M (BSA <1.8)
			48 ± 3.6 high impact F (BSA <1.5)
			51.2 ± 3 low impact M (BSA <1.8)
			45.3 ± 2.8 low impact F (BSA <1.5)
	IVS (mm)	Boraita *et al*. [75], Spain – 2022 (3282 elite athletes, mean age 23 ± 6 years)	9 ± 1 M
			8 ± 1 F
		Magalski *et al*. [76], USA – 2011 (964 competitive athletes, aged 18–21 years)	9 ± 1 M
			8 ± 1 F
			9 ± 1 white
			9 ± 1 black
		D’Andrea *et al*. [78], Italy – 2010 (615 elite athletes, mean age 28.4 ± 10 years)	9.7 ± 3.1 endurance
			9.2 ± 2.1 strength
	Systolic function (EF%)	Boraita *et al*. [75], Spain – 2022 (3282 elite athletes, mean age 23 ± 6 years)	61 ± 7 M and F
		D’Andrea *et al*. [78], Italy – 2010 (615 elite athletes, mean age 28.4 ± 10 years)	69.7 ± 4.7 endurance
			67.1 ± 3.8 strength
	Diastolic function (E/A)	Boraita *et al*. [75], Spain – 2022 (3282 elite athletes, mean age 23 ± 6 years)	85 ± 14/43 ± 11 M
			92 ± 14/45 ± 13 F
LA	Antero-posterior diameter (mm)	Boraita *et al*. [75], Spain – 2022 (3282 elite athletes, mean age 23 ± 6 years)	35.9 ± 4.7 M
			32.1 ± 4.2 F
		Magalski *et al*. [76], USA – 2011 (964 competitive athletes, ages 18–21 years)	34 ± 4 M
			30 ± 4 F
			32 ± 4 white
			33 ± 4 black
		D’Andrea *et al*. [78], Italy – 2010 (615 elite athletes, mean age 28.4 ± 10 years)	34.5 ± 5.5
	Longitudinal diameter (mm)	Boraita *et al*. [79], Spain – 2016 (3281 elite athletes, mean age 23.1 ± 5.7 years)	52.6 ± 5.9 M
			48.1 ± 5.5 F
	Area (cm)	Gjerdalen *et al*. [80], Norway – 2015 (595 elite athletes, mean age 25.1 ± 4.6 years)	20.7 ± 4.4
	Volume index (mL/$m^{2}$)	D’Andrea *et al*. [78], Italy – 2010 (615 elite athletes, mean age 28.4 ± 10 years)	28.2 ± 9.2 M
			26.5 ± 7.2 F

LV, left ventricle; LA, left atrium; BSA, body surface area; EF, ejection fraction; F, female; LA, left atrium; LAVI, left atrial volume index; LV, left ventricle; EDD, left ventricular end-diastolic diameter; M, male; IVS, interventricular septum; E/A, early (E) to late (A) diastolic filling velocity.

**Table 3.** **Athlete’s right heart echocardiography evaluation**.

Cardiac chamber	Parameter	Mean value (95% CI)	Mean value (95% CI)	Mean (± SD) F athletes	Study
		M endurance athletes	M strength athletes		
RV	RVOT PLAX (mm)	29 (26–33)	29 (26–33)	28 ± 2	D’Ascenzi *et al*. [81], Italy – 2017 (6806 competitive athletes, aged 18–39 years)
	RVOT PSAX (mm)	34 (32–35)	34 (32–35)	30 ± 1	
	Basal diameter (mm)	40 (38–42)	38 (31–45)	35.7 ± 0.2	
	Midcavity diameter (mm)	29 (27–30)	26 (23–29)	29.1 ± 0.3	
	RV wall thickness (mm)	4.2 (3.9–4.4)	4.0 (3.5)		
	End-diastolic area (${\mathrm{cm}}^{2}$)	23 (20–27)	21 (17–25)	23.0 ± 0.1	
	End-systolic area (${\mathrm{cm}}^{2}$)	13 (10–15)	10 (8–13)		
	TAPSE (mm)	25 (22–28)	25 (22–28)		
	FAC (%)	35 (32–38)	41 (32–49)	39 ± 4	
RA	Antero-posterior diameter (mean ± SD, mm)	45.1 ± 5.8			Gjerdalen *et al*. [80], Norway – 2015 (595 elite athletes, mean age 25.1 ± 4.6 years)
	Area (${\mathrm{cm}}^{2}$)	18 (14–23)	18 (14–23)	16 ± 1	D’Ascenzi *et al*. [81], Italy – 2017 (6806 competitive athletes, aged 18–39 years)

F, female; FAC, fractional area change; M, male; RA, right atrium; RV, right ventricle; RVOT, right ventricular outflow tract; TAPSE, tricuspid annulus peak systolic excursion; PLAX, parasternal long axis; PSAX, parasternal short axis.

While a mild dilatation of the aorta can sometimes be observed, particularly in some categories of athletes (i.e., master endurance athletes), a more-than-mild dilatation is not part of the athlete’s heart and should warrant further investigations in case of an aortic root more than 40 mm in males (an indexed values of 20 mm/$m^{2}$ for allometric scale) and 34 mm in females and more than 34 mm for the proximal ascending aorta [65, 73].

**Fig. 3.**
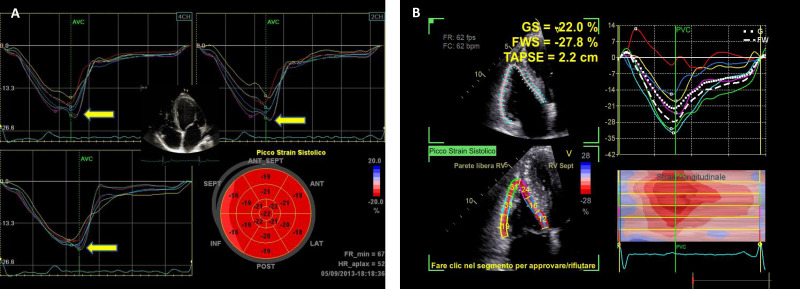
**Global longitudinal strain (GLS) values of the left ventricle (A) and right ventricle (B) in a professional athlete: the bull’s eye is within normal values despite left ventricular hypertrophy (A) and right ventricular dilation (B)**. GS, global strain; FWS, free-wall strain; TAPSE, tricuspid annular plane excursion.

In the last decades, advances in ultrasound technology have evolved echocardiography from simple M-mode to 2-dimensional imaging, Doppler assessments, 3-dimensional (3D) anatomical imaging, and dimensional analysis of myocardial deformation [84]. Speckle-tracking echocardiography is very useful in evaluating the athletes’ heart [85], especially in identifying regional wall motion abnormalities and pre-clinical impairment in HCM and DCM. The mean value of global longitudinal strain in athletes is –18.1 ± 2.2% in LV and –27 ± 6% in RV [65] (Fig. 3). Three-dimensional echocardiography has added quantitative information to assess the athlete’s heart; cardiac volumes and mass can be estimated more precisely than 2D echo without geometric assumptions [86]. Moreover, myocardial work, calculated by adjusting myocardial deformation to the instantaneous LV pressure, has been recently proposed to have a role in the athlete’s heart diagnostic process, due to its less dependency on loading contraction than global longitudinal strain [87].

#### 3.2.2 Exercise-Stress Test

EST is the most widely available functional test. The continuous ECG and blood pressure (BP) monitoring of the subject during a treadmill or cycle-ergometer incremental test, provides information on exercise capacity, heart rate and BP response to exercise, other than exercise-induced abnormalities, including arrhythmias [88]. It can be used for diagnostic, prognostic, or functional evaluation purposes [89], and it can also be adopted using different protocols based, for instance, on the type of sports practiced by the athlete [65]. Indeed, both treadmill and cycle ergometer protocols have their strengths and disadvantages and should be used with a precise aim in athletes [90]. However, some contraindications to EST must be considered [91]. In addition, EST requires specific care because of the wide range of normal findings, the use of different stress-inducing protocols, and the lack of generally accepted reference values [92].

In athletes over 35 years of age, EST investigates the presence of a silent ischemic cardiovascular disease through specific alterations in ST-segment and T-wave. However, it is less specific for myocardial ischemia than other functional tests, especially in asymptomatic and low-risk individuals [8]: for example, an asymptomatic upsloping ST-segment depression with normalization in the early (<1 min) phase of recovery should not be considered pathological [93].

EST also permits assessing BP changes during exercise. An exaggerated BP response to exercise should lead to starting or optimizing antihypertensive medical therapy and performing a cardiologic evaluation, even if the athlete is normotensive at rest since it predicts incident and early hypertension in athletes [94]. In a large cohort of elite athletes undergoing EST, the 95th percentile of BP values was 220/85 mmHg in males and 200/80 mmHg in females [95]. Also, a decrease in BP during the test is not normal and should be further investigated.

Even if ventricular arrhythmias may be unrelated to heart diseases, some of them could be a marker for an arrhythmogenic condition in athletes with no relevant history, normal physical examination, and resting ECG, and therefore EST plays a pivotal role in describing its effort-related characteristics [96]. Other arrhythmias that can be studied through EST are atrial fibrillation, first- or second-degree AV block, and asymptomatic pre-excitation [65]. Also, QT interval adaptation to exercise and recovery phase is an important phenomenon to consider when evaluating an EST.

#### 3.2.3 24-Hours ECG Holter Monitoring

ECG continuous monitoring is a method that provides more information for the detection of cardiac rhythm alterations than resting 12-lead ECG recording. ECG Holter monitoring recording with 12-leads configuration should always be preferred to determine the origin of ventricular arrhythmias (morphology and axis) and the presence of ischemia [97]. The monitoring period is usually 24 hours, even if it may be longer in specific cases, and should always include a training session, to reproduce as much as possible the “natural” physical effort of the athlete: this allows to study the response of the arrhythmias to exercise and to elicit arrhythmias that are in relation with the effort. However, ECG Holter monitoring in athletes is often rich in motion artifacts; therefore, an experienced physician is required to interpret it.

Life-threatening arrhythmias are infrequent among young athletes who require ECG monitoring, whereas their presence may suggest an underlying cardiac disease according to some specific characteristics [98] (Table 4, Ref. [96, 97]). A diary in which the patient reports the main daily activities (i.e., exercise sessions, sleeping times, etc.), any drug therapy taken or symptoms experienced should always be part of the assessment.

**Table 4.** **Features of uncommon premature ventricular beats in athletes that should raise suspicious of underlying disease requiring further investigations [96, 97]**.

Characteristics of uncommon PVBs	
Ectopic QRS morphology	RBBB and wide QRS (≥130 ms)
	LBBB with intermediate or superior axis
Response to exercise testing	Persistence/increase
Complexity of PVBs	Couplets, triplets or NSVT
	Polymorphic
Short coupling interval*	Yes

*: PVBs are superimposed on the preceding T-wave peak or earlier (i.e., R on T). LBBB, left bundle branch block; PVBs, premature ventricular beats; RBBB, right bundle branch block; NSVT, non-sustained ventricular tachycardia.

Progress in science and technology has led to the development of numerous devices, such as the external loop recorder, event recorders or wearables, for assessing cardiac arrhythmias, that are nowadays available for patients and should be used in selected cases (i.e., symptomatic athletes with infrequent symptoms) and with careful interpretation [99, 100].

#### 3.2.4 Cardiopulmonary Exercise Test

CPET is a valuable tool to evaluate the responses of the cardiac, pulmonary, vascular, and musculoskeletal systems to exercise [101, 102, 103, 104]. Although still underutilized, its high reproducibility offers important prognostic and diagnostic information [105] and can be integrated with other imaging techniques [106]. Different from an EST, CPET involves measurements of respiratory oxygen uptake, carbon dioxide production, and ventilatory measures during a symptom-limited exercise test. CPET indications in athletes are manifold, including cardiorespiratory fitness estimation, evaluation of symptoms of unexplained origin and exercise prescription [107]. It is known that highly trained athletes have higher cardiorespiratory fitness compared to untrained individuals or low-trained athletes [108]. Therefore, cardiorespiratory fitness considered in the normal predicted ranges may mask latent disorders or physiological impairments in athletes. For this reason, interpreting CPET results requires caution within the clinical context, as predicted gas exchange parameters have been derived in the general population [109]. Moreover, athletes show further differences in exercise hemodynamic response and gas exchange parameters compared to non-athletes, including higher cardiac output, faster heart rate recovery, higher prevalence of exercise-induced arterial hypoxemia, and lower breathing reserve [107, 110] (Table 5, Ref. [107, 110, 111, 112]), even if reference values have yet to be determined [113]. Knowing these parameters in the context of the athlete’s physiological response to exercise could help guide the differential diagnosis between the athlete’s heart and underlying CV diseases [114].

**Table 5.** **Expected CPET parameters in healthy individuals and their response to exercise in athletes**.

CPET Parameter	In healthy subjects [111, 112]	In elite Athletes [107, 110]
HR max	≥85% age-predicted HR max	Equal or more
	HR increase 10 bpm per every 3.5 mL/kg/min of ${\mathrm{VO}}_{2}$	
HR recovery	>12 beats at first-minute recovery	More
Blood pressure	SBP increase 10 mmHg per every 3.5 mL/kg/min of ${\mathrm{VO}}_{2}$	More
	DBP stable or fall	
${\mathrm{SpO}}_{2}$	${\mathrm{SpO}}_{2}$ ≥95% (rest and exercise)	Equal or less
	${\mathrm{SpO}}_{2}$ should not decrease below 95%	
${\mathrm{VO}}_{2}$ peak	Percent predicted values should be about 100%	Quite more
		Predictive equations for endurance athletes [110]
		➢ Treadmill: ${\mathrm{VO}}_{2}$ peak (L/min) – 0.83(sex) + 0.033(height) – 0.017(age) – 1.15
		➢ Cycle: ${\mathrm{VO}}_{2}$ peak (L/min) – 0.72(sex) + 0.048(height) – 0.00019(${\mathrm{age}}^{2}$) – 4.30
${\mathrm{VO}}_{2}$ at VT	Not mentioned	Quite more
Oxygen pulse	Percent predicted values should be about 100%	Quite more
	Continual linear rise throughout the exercise with possible plateau approaching maximal exertion	
Breathing reserve	>20%	Less
VE/${\mathrm{VCO}}_{2}$ slope	<30 throughout the exercise	No differences
${\mathrm{PETCO}}_{2}$	Not mentioned for apparently healthy individuals (usually resting ${\mathrm{PETCO}}_{2}$ is between 36 and 42 mmHg)	No differences
OUES	Not mentioned	No differences

CPET, cardiopulmonary exercise test; HR, heart rate; SBP, systolic blood pressure; DBP, diastolic blood pressure; ${\mathrm{SpO}}_{2}$, peripheral oxygen saturation; ${\mathrm{VO}}_{2}$ peak, oxygen consumption at peak exercise; ${\mathrm{VO}}_{2}$ at VT, oxygen consumption at the ventilatory threshold; VE/${\mathrm{VCO}}_{2}$ slope, minute ventilation/carbon dioxide production slope; ${\mathrm{PETCO}}_{2}$, partial pressure of end-tidal carbon dioxide; OUES, oxygen uptake efficiency slope.

In clinical settings, CPET is generally used to evaluate the etiology of unexplained symptoms such as exertional dyspnea, chest discomfort, and fatigue. Moreover, excessive training load without an adequate recovery period exposes athletes to decreased performance and sometimes even to overtraining syndrome. CPET may be particularly useful in this condition.

Finally, CPET can be used for a tailored exercise prescription, not only to improve the performance of elite endurance athletes but also in patients at risk of and with CV disease, especially those who are older and engage for the first time in moderate to vigorous physical activity [111, 115]. Through the identification of ventilatory thresholds, the physician may draw out a personally tailored program with the appropriate level of intensity associated with possible enhancements for healthy athletes and proven benefits for patients with chronic diseases [116, 117]. Moreover, CPET should be part of the routine assessment of patients with cardiomyopathies who wish to exercise to obtain information about functional capacity and risk stratification [97, 106, 118, 119].

### 3.3 Third-Line Evaluation

In the presence of abnormal, uncertain, and/or controversial findings from the upstream diagnostic work-up (first- and second-line evaluation), other CV diagnostic modalities can be useful to differentiate between physiological and pathological adaptation of the athlete’s heart. However, due to their high cost and limited availability, these are not routinely recommended, but must be guided by a precise clinical suspicion, carefully considering each indication (Table 6). While CMR is the contemporary gold standard for defining myocardial structure and myocardial tissue architecture and is increasingly applied both for the study and clinical management of athlete’s heart, stress imaging represents a useful tool to unmask reduced cardiac functional reserve and covert pathological changes that are not evident at rest, especially in athletes in whom arrhythmias and/or early-stage cardiomyopathies are suspected [3]. In that sense, ESE represents the first choice, but also CCT and nuclear CV imaging techniques have pivotal diagnostic importance, especially in specific populations, such as master athletes. Finally, the use of genetic testing in athletes is increasing because genetic studies have identified many genetic variants that underpin cardiac disorders and technological advances have transformed genetic testing into a more readily available and affordable clinical tool [120].

**Table 6.** **Details of some third-line cardiac diagnostic techniques in athletes**.

Diagnostic techniques	Pros	Cons
ESE	- Assessment of biventricular function during exercise	- Require specific and expensive equipment
	- Unmask pathologies not apparent at rest	- Motion artefacts
	- Physiological activation of the cardiovascular system	- Limiting skeletal muscle fatigue in individuals not accustomed to cycling
	- Diastolic stress testing	
	- Ability to characterize valve function and morphology	
	- Non-radiation imaging modality	
	- Low cost	
CMR	- Non-radiation imaging modality	- Costs
	- High spatial and temporal resolution	- Limited access
	- No blind spots	
	- Not limited by the thoracic wall, pulmonary parenchyma or wall thickness evaluation	
	- Accurate evaluation of cardiac function, flow, volumes and perfusion	
	- Excellent evaluation of wall motion abnormalities	
	- Multiparametric tissue characterization (LGE, mapping techniques)	
CCT	- High spatial resolution	- Costs
	- Obtain high-quality multiplanar reconstructions in any desired image orientation	- Limited access
	- Low contrast volume and low radiation dose	- Radiation dose
	- Evaluate morphological patterns and global and regional kinetic functions	- Low temporal resolution
	- Short examination time	

CMR, cardiac magnetic resonance; ESE, exercise stress echocardiography; LGE, late gadolinium enhancement; CCT, cardiac computed tomography.

#### 3.3.1 Exercise-Stress Echocardiography

ESE is a reliable, safe, non-invasive imaging test that provides a dynamic cardiac function evaluation. Combined with clinical and ECG data, ESE helps detect cardiac abnormalities that may not occur at rest, such as exercise-induced ischemia in athletes with suspected coronary artery disease or congenital coronary artery anomalies [121]. Furthermore, ESE can assess contractile reserve during exercise in endurance athletes with LV and/or RV dilatation and mildly reduced ejection fraction at rest: an increase of LV EF of at least 15% during exercise may support the diagnosis of athlete’s heart [122]. Finally, ESE may be useful in athletes with valvular heart disease, providing information about exercise tolerance, biventricular contractile reserve, changes in hemodynamics (LV filling pressure, pulmonary pressure), and valvular functional parameters (transvalvular gradients, regurgitation entity—i.e., bicuspid aortic valve) [78].

Pharmacological stress is generally not indicated in athletes and an exercise test is usually performed through a bed cycle ergometer. Since the time for image acquisition is limited, the echocardiographic protocol is usually tailored to the clinical indication [65]. However, even if some limitations to the use of ESE exist, improvements in imaging equipment and technology, and allowing the movement towards more robust quantitative analysis, have led ESE to become a valuable tool in the diagnostic process of athlete’s heart.

#### 3.3.2 Cardiovascular Magnetic Resonance 

CMR is an established imaging modality for the cardiovascular assessment of athletes. It is a third-tier diagnostic tool that helps to discriminate between physiology and pathology [65], and it is superior to echocardiography in differentiating athlete’s heart from structural and functional change [123]. The limitations of CMR include, among others, high cost, limited accessibility and claustrophobia, other than untested or low interobserver variability [124].

**Fig. 4.**
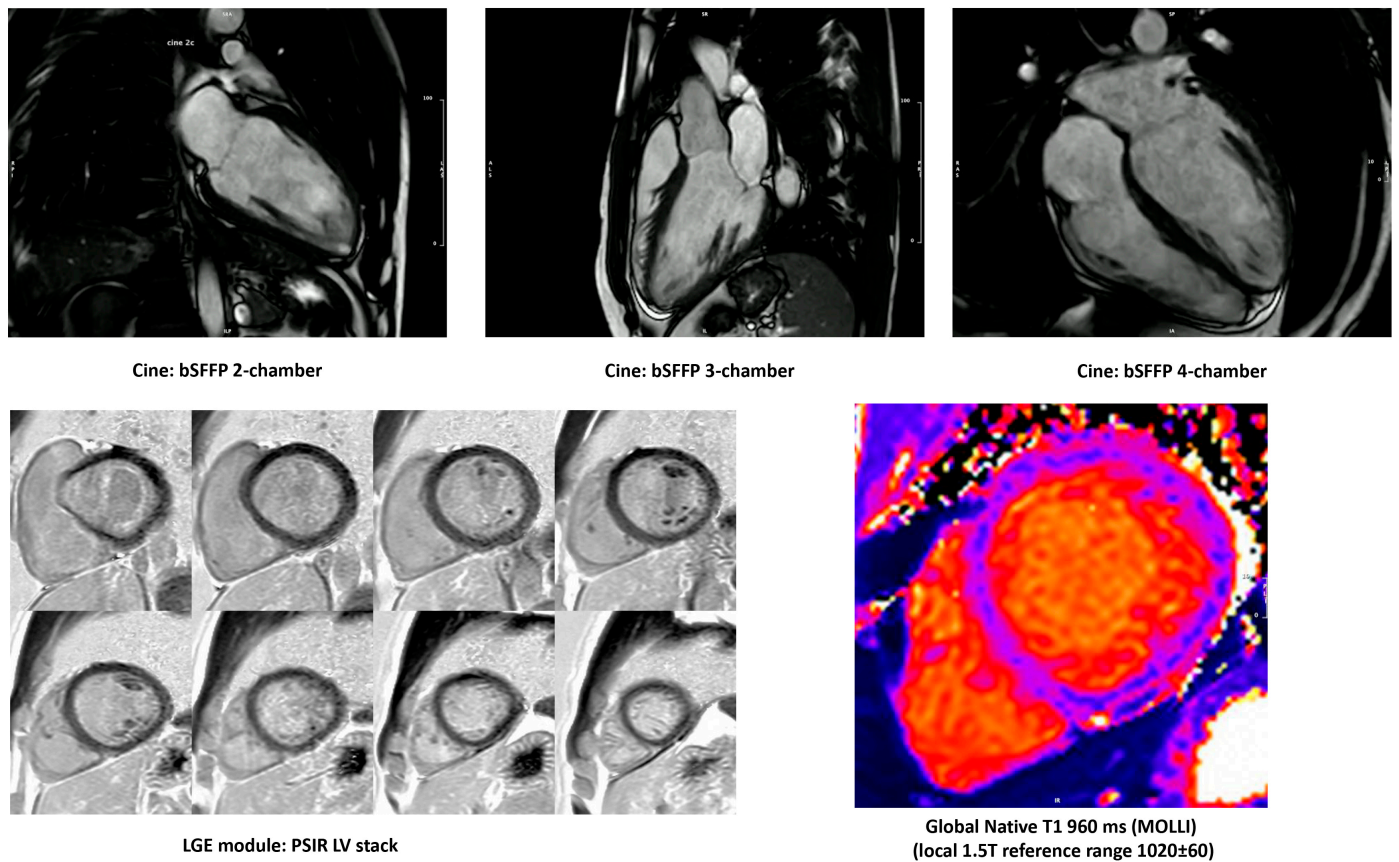
**Cardiovascular magnetic resonance of an endurance athlete, investigated for premature ventricular beats and left ventricle (LV) dilatation and demonstrating balanced LV dilatation, borderline-low normal LV ejection fraction, no regional wall motion abnormalities, high-normal stroke volume, no late gadolinium enhancement, low-normal native myocardial T1, normal extracellular volume (25%)**.

CMR is the gold standard for defining biventricular volumes and mass, and quantification of volumes and flow (Table 7, Ref. [125]) [126], providing advanced myocardial tissue characterization with excellent accuracy and precision. CMR has the incremental benefit of allowing tissue characterization by identifying myocardial inflammation and fat infiltration through T1 and T2 weighted images and mapping. CMR allows the detection of replacement fibrosis by late gadolinium enhancement (LGE) imaging, also pointing to the description of ischemic vs nonischemic patterns of myocardial damage [127]. As such, CMR supports the diagnosis of myocarditis and cardiomyopathies [128], such as HCM [129] and ACM [130, 131, 132, 133]. To differentiate between pathologic modification and physiologic remodeling, cardiac volumes and masses should always be compared to reference ranges deriving from CMR studies on healthy athletes [134], and adjusted to several factors, including type of sport, static and dynamic component, training hours per week, body surface area, age, gender, and ethnicity [135] (Fig. 4).

**Table 7.** **Normative CMR values for male endurance athletes**.

Cardiac chamber	Parameter	Mean (95% CI)	Study
LV	EDV (mL)	208 (195–220)	D’Ascenzi *et al*. [125], Italy – 2019 (1053 competitive athletes, aged 18–55 years)
	EDV index (mL/$m^{2}$)	111 (104–121)	
	ESV (mL)	74 (68–79)	
	ESV index (mL/$m^{2}$)	49 (45–55)	
	SV (mL)	125 (116–135)	
	SV index (mL/$m^{2}$)	63 (45–79)	
	EF (%)	59 (58–61)	
RV	EDV (mL)	230 (214–245)	
	EDV index (mL/$m^{2}$)	120 (113–126)	
	ESV (mL)	101 (91–110)	
	ESV index (mL/$m^{2}$)	55 (49–61)	
	SV (mL)	123 (112–134)	
	SV index (mL/$m^{2}$)	65 (59–71)	
	EF (%)	54 (52–56)	

CMR, cardiac magnetic resonance; LV, left ventricle; RV, right ventricle; EDV, end-diastolic volume; ESV, end-systolic volume; SV, stroke volume; EF, ejection fraction.

In the last years, it is spreading the use of stress CMR that has the advantages to assess biventricular function, wall motion and valve function during exercise, even if it requires high levels of training, dedicated devices, and long-scan times [136].

#### 3.3.3 Coronary Computed Tomography 

CCT shows high accuracy in evaluating coronary atherosclerosis and coronary origin and course [121]. The assessment of coronary arteries by CCT is non-invasively performed and requires a very low radiation dose, thanks to the latest generation scanners (from 0.7 to 1 mSv with optimized acquisition parameters and protocols [136]). Therefore, depending on the local availability and expertise, CCT may be considered in athletes with symptoms suggestive of CAD and in older, asymptomatic athletes with risk factors for CV disease or equivocal exercise stress test. Indeed, it has been recently theorized the use of CCT in the screening process of mature athletes increases the negative predictive value for excluding coronary artery disease [137]. Moreover, CCT should be considered when a precise definition of proximal coronary anatomy or characterization of great vessel morphology is indicated [136]. When dilatation of the aortic root or ascending aorta is suspected, at least one comprehensive aortic tomographic assessment by CT angiography or angio-MR should be performed [73]. Cardiac CT well visualizes pericardial thickening and calcification, and CT attenuation values may differentiate pericardial fluid contents. Once anatomical abnormalities have been detected, a CV functional assessment performed during exercise is required to evaluate their functional clinical impact.

However, due to ionizing radiation exposure and high costs, this imaging modality is not recommended as the first-line technique for young athletes.

#### 3.3.4 Nuclear Imaging Techniques 

Myocardial perfusion scintigraphy techniques are generally considered a valuable diagnostic and prognostic modality and often used for further diagnostic evaluation in athletes with electrocardiographic findings indicative of myocardial ischemia in the PPS [138]. SPECT or PET can research exercise-induced ischemia and stratify the risk of athletes with suspected or known CAD, anomalous origin, or course of coronary arteries (e.g., myocardial bridging) [139]. The accuracy of both PET and SPECT in detecting CAD is excellent [140]. However, PET may be preferred in balanced 3-vessel disease since it permits absolute quantification of myocardial blood flow. Conversely, SPECT can only provide semi-quantitative values (normalized to the maximum value), failing to detect relative perfusion differences [141, 142]. Moreover, even SPECT specificity in competitive athletes has to be considered reduced, given that myocardial perfusion defects can be present also in healthy young male athletes, and they are associated with LV hypertrophy and no wall motion abnormalities on echocardiography [142]. Thus, cardiac nuclear imaging in the athlete’s setting is more suitable for research purposes than for a clinical application and should not be recommended as a first-line test in competitive athletes [123]. According to European guidelines [8], nuclear imaging may also be considered an alternative or complementary exam to ESE or CCT for evaluating asymptomatic individuals aged >35 years with CV risk factors before engaging in high or very high-intensity sports.

#### 3.3.5 Genetic Testing in Athletes

Genetic testing is a valuable tool for diagnosing several inherited cardiac disorders [120, 143]. In athletes, it can be beneficial in terms of diagnosis, management, decisions relating to sports participation, and prognosis [144]. Moreover, identifying disease-causing mutations allows cascade screening in first-degree family members due to the autosomal dominant pattern in most inherited cardiac disorders [145].

The diagnostic yield of genetic testing is significantly different according to the clinical phenotype of the athlete. From a general point of view, genetic testing in diagnosing an inherited cardiac disorder is useful in individuals with clear phenotypes. In recent years, attention has been given to the diagnostic role of genetic testing in an individual who exhibits an overlapping phenotype between inherited cardiac disease and athlete’s heart [146, 147]. In selected cases, when a comprehensive clinical evaluation is suspicious but fails to reach a definitive diagnosis of inherited cardiac disease, genetic testing may be considered, keeping in mind the specific diagnostic yield for each disease and that it can be even lower in athletes. The benefit of genetic testing should always be weighed with potential harm. The genetic testing panel should only include genes with supporting solid evidence to cause the athletes’ clinical phenotype to minimize the identification of a variant of uncertain significance or allelic variants associated with the different clinical phenotype [148], which increases the difficulties inherent to the interpretation of genetic testing results.

Since the diagnostic yield of genetic testing is significantly different according to the clinical phenotype of the athlete, physicians involved in the athlete’s management should have a solid understanding of the indications, strengths and limitations of genetic testing (Table 8, Ref. [120]).

**Table 8.** **Indication of genetic testing in competitive athletes [120]**.

Genetic test recommendations	Pre-test probability	ECG abnormalities
Recommended	High	HCM
		DCM
		ACM
		LQTS
		CPVT
May be recommended	Intermediate	LVH + additional features
		LV + additional features
		RV dilatation + additional features
		QTC >480 ms + additional features
		NSVT or polymorphic PVC + additional features
Not recommended	Low	Isolated LVH
		Isolated LV dilatation
		Isolated RV dilatation
		Isolated QT prolongation
		Isolated monomorphic PVC
		Isolated T-wave inversion

ECG, electrocardiography; LVH, left ventricle hypertrophy; LV, left ventricle; RV, right ventricle; PVC, premature ventricular contraction; NSVT, non-sustained ventricular tachycardia; HCM, hypertrophic cardiomyopathy; DCM, dilated cardiomyopathy; ACM, arrhythmogenic cardiomyopathy; LQTS, long QT syndrome; CPVT, catecholaminergic polymorphic ventricular tachycardia.

## 4. The Grey Zones in the Athlete’s Heart

Athlete’s heart is characterized by cardiac remodeling features that can resemble those found in pathological conditions. Distinguishing athletic cardiac remodeling from cardiomyopathy is a frequent clinical dilemma for physicians evaluating an athlete [3, 72]. There are, in fact, several “grey zones” in which physiology and pathology overlap, and therefore it is essential to relate the degree of cardio-circulatory adaptations of athletes to the biomechanical characteristics of the practiced sport [122, 149]: LV wall thickening, LV dilatation, RV dilatation and LV hypertrabeculation (Table 9). It is, therefore, necessary to have a precise definition of the features of the athlete’s heart and stringent criteria to optimize the clinical management of these subjects to be able to make a differential diagnosis with HCM [150], DCM [151], left ventricular noncompaction (LVNC) and ACM.

**Table 9.** **Differential diagnosis between athlete’s heart and SCD-related cardiomyopathies in diagnostic grey zones**.

	LV wall thickening	LV dilatation	RV dilatation	LV hypertrabeculation
Athlete’s heart findings	∙ Strength and mixed disciplines (more common)	∙ Endurance athletes (typically)	∙ Endurance and mixed disciplines (more common)	∙ Afro-Caribbean ethnicity
	∙ Afro-Caribbean ethnicity (more common)	∙ Asymptomatic	∙ Asymptomatic	∙ Asymptomatic
	∙ Male gender (more common)	∙ Unremarkable family history	∙ Unremarkable family history	∙ Unremarkable family history
	∙ Normal SBP	∙ Normal ECG	∙ Normal ECG	∙ Normal ECG
	∙ Asymptomatic	∙ Sometimes association with mild reduction in LVEF with normal function during the exercise		
	∙ Unremarkable family history			
	∙ Normal ECG			
	∙ Mild symmetric and balanced LVH (often reversible after detraining)	∙ Concomitant RV dilatation and/or mild LVH	∙ RV dilatation often reversible after detraining	∙ Normal LV systolic function
	∙ Concomitant absence of a small LV cavity size	∙ Preserved/mildly reduced LVEF with normal function during the exercise	∙ Absence of wall motion abnormalities	∙ Normal LV GLS
	∙ Normal/supranormal LV diastolic function	∙ Normal LV GLS	∙ Concomitant LV dilatation	∙ Normal/supranormal LV diastolic function
	∙ Preserved LV systolic function	∙ Normal/supranormal LV diastolic function	∙ Normal RV morphology	∙ Normal or increased compacted LV wall thickness
	∙ Normal LV GLS	∙ Normal/mildly enlarged LA and RA	∙ Preserved or mildly reduced LVEF with normal function during the exercise	
		∙ Normal aortic and mitral valves	∙ Normal LV GLS	
			∙ Normal/supranormal LV diastolic function	
			∙ Normal/mildly enlarged LA and RA	
			∙ Normal RV systolic function	
			∙ Normal RV GLS	
			∙ Normal sPAP or eventually upper limits	
			∙ Normal tricuspid and pulmonary valve	
Suspicious findings	∙ Isolated/asymmetric LVH (not reversible with detraining)	∙ Reduced LV systolic function	∙ RV dilatation not reversable with detraining	∙ Compacted layer <5 mm
	∙ LV diastolic disfunction	∙ LV diastolic disfunction	∙ Reduced LV systolic function	∙ Reduced LV systolic function
	∙ Other anatomic abnormalities (mitral valve leaflet elongation, anomalous papillary muscle insertion, myocardial crypts or recesses)	∙ Presence of wall motion abnormalities	∙ Reduced RV function	∙ LGE on CMR
	∙ Exercise-induced LVOT or mid-cavity obstruction on ESE	∙ Impaired contractile reserve during ESE or stress CMR	∙ RV morphology abnormalities (sacculations, aneurysms, and focal thinning)	
	∙ LGE/fibrosis on CMR		∙ Presence of wall motion abnormalities	
			∙ Impaired contractile reserve during ESE or stress CMR	
Differential diagnosis	∙ HCM	∙ DCM	∙ ACM	∙ LVNC
	∙ Hypertensive heart	∙ Toxic CMP	∙ Toxic CMP	∙ Recent pregnancy
	∙ Anabolic steroid abuse	∙ Myocarditis	∙ Pulmonary hypertension	∙ Sickle cell disease
	∙ Infiltrative heart disease	∙ Nutritional deficiency	∙ CHD	∙ Aortic/mitral regurgitation
	∙ Valvulopathy	∙ Tachyarrhythmias-mediated CMP	∙ Valvulopathy	
		∙ Valvulopathy		

HCM, Hypertrophic cardiomyopathy; DCM, dilated cardiomyopathy; LVNC, left ventricular noncompaction; ACM, arrhythmogenic cardiomyopathy; LVEF, left ventricle ejection fraction; LV, left ventricle; LA, left atrial; GLS, global longitudinal strain; RV, right ventricle; LGE, late gadolinium enhancement; CMR, cardiac magnetic resonance; CMP, cardiomyopathy; LVH, left ventricle hypertrophy; CHD, congenital heart disease; sPAP, systolic pulmonary artery pressure; ESE, exercise stress echocardiography; LVOT, left ventricle outflow tract; SBP, systolic blood pressure; SCD, sudden cardiac death; ECG, electrocardiography.

It must be emphasized that the effective use of clinical imaging data requires integration with other aspects of the clinical presentation, including the presence or absence of symptoms, a family history of genetic heart disease or SCD, the 12 lead ECG and maximal exercise testing. Therefore, the choice between the proposed step-by-step approach must always be guided by the clinical suspicions, considering the entire clinical scenario the entire spectrum of CV diseases that can afflict the athlete (Table 10).

**Table 10.** **Practical approach to the athlete’s heart diagnosis**.

1st-line screening	2nd-line screening	Clinical suspicious	3rd-line screening	
History + physical examination + ECG	Echocardiography + EST/CPET + 24-hours ECG Holter		First choice	Second choice
		Cardiomyopathies	CMR	Genetic testing
		CAD	Echo-stress	CCT, SPECT or PET
		Valvulopathies	CMR	Echo-stress
		Myocarditis, pericarditis	CMR	
		Coronary artery abnormalities	CCT	
		Aorthopathies	CCT	
		Channelopathies	Genetic testing	

CAD, coronary artery disease; ECG, electrocardiogram; EST, exercise stress test; CPET, cardiopulmonary exercise test; CMR, cardiac magnetic resonance; CCT, cardiac computer tomography; SPECT, single photon emission computer tomography; PET, positron emission tomography.

## 5. Conclusions

Discriminating the athlete’s heart from the differential diagnosis of early-phenotype cardiomyopathy or a concealed cardiovascular pathology requires a comprehensive diagnostic work-up based on morphologic, electrical, structural, and functional evaluations. Since the wide availability and the indications of several multimodality techniques, a practical step-by-step approach is helpful to systematically proceed in the evaluation, if indicated after the first-line screening PPS, only if second- and third-line diagnostic modalities are needed.

Despite reducing the false-positive rate, many athletes inevitably fall into the grey zone with multiple layers of overlap between pathology and physiologic remodeling. A multimodality cardiovascular diagnostic approach can play a central role in supporting an appropriate final diagnosis.
